# Lifespan of super-alloy Waspaloy cutting tools

**DOI:** 10.1016/j.heliyon.2019.e01388

**Published:** 2019-04-16

**Authors:** Shao-Hsien Chen, Yu-Lun Ho

**Affiliations:** a The Graduate Institute of Precision Manufacturing, National Chin-Yi University of Technology, Tai-Chung, Taiwan, China; b Department of Mechanical Engineering, National Chin-Yi University of Technology, Tai-Chung, Taiwan, China

**Keywords:** Materials science, Mechanical engineering

## Abstract

An airplane has about 6-million components and parts, mainly the engine, undercarriage, constructions and so on. Among them, nickel base materials are widely used, including engine, cartridge receiver, compressor drum and other industries application, such as energy, petrochemical industry, mould, etc. Nickel base alloy, with anti-corrosion and thermo stability, has good mechanical properties under high temperature. Since 1950s, due to the development of precision casting technology, a series of cast nickel base super-alloy with high strength has been developed, namely the super-alloy complying with the above conditions. In recent years, with the changing of military and civil aerospace, the usage amount of nickel base super-alloy also increases along with the date. This research mainly used Waspaloy of nickel base material to do cutting research and uses regression analysis to find significant factor of cutting tool's life, and performs the optimization experiment. The cutting research mainly uses TiAlN to coat cutting tools. In the conclusion, it discusses main factors that influence the tool life, such as the cutting speed, depth, feed rate and so on. Finally it establishes tool life formula with regression analysis method, in this case when cutting speed V = 30.77 m/min and cutting depth dp = 0.0367mm, the minimum wear prediction can be obtained.

## 1 Introduction

**Fig. 1:**
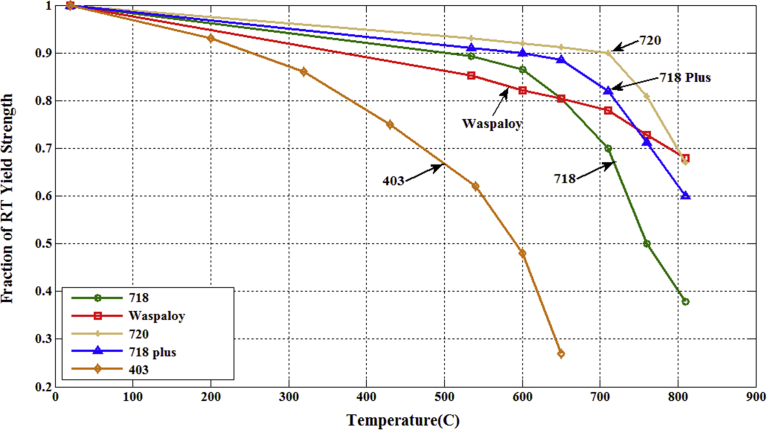
The effect of temperature on the tensile yield strength of selected nickel-based alloys [6].

Recent industry and research used semi-circular inductor to heat the titanium alloy Ti-5553 [12]. In the experiment, it was found that the cutting force at 500 °C decreased by about 13%, and the cutting force can decrease by 34% when the temperature is 750 °C. Thus it can be seen that the cut ability had obvious difference and importance when there is no additional heat source to preheat, while the performance of cutting tool is very important during the cutting processing, On the 650–750 °C has the increase of temperature will cause decreasing strength, as shown in Fig. 1 [6]. The abrasion of cutting tools will direct influence processing efficiency and precision. So it is considerably important to predict the life of cutting tools accurately [1, 2, 3]. Response Surface Methodology (RSM) can be applied to WC-Co cutting tool without coating. In the life establishment of Titanium alloy Ti-6AL-4V cutting tool, dry cutting and end mill were used. From the cutting tool life model, it can be found that cutting speed, depth, and feed rate were 100%, the ratio influencing the tool life were 70%, 27% and 37% [4].

Response Surface Methodology (RSM) uses statistical method to assist to solve the relation between multi independent variables and certain response variable in the unknown function, which combine experimental design and regression analysis, and through the experimental design method, in the space we set to input parameter, and get the required response value and its corresponding input parameter position. So the graphical method is used to present the RSM, and the purpose of which is to provide a set of effective analysis and solution method to optimize the product design, processing and system and other problems. In the application, the following two restrictions exist: 1. It is suitable for continuous system, which is based on the assumption that the measurement scale of response value and independent variable is continuous; 2. The independent variable which affects the system is measurable (controllable and uncontrollable variable). Under the assumption of RSM and the restrictions of application system, the best experimental value or task variable value can be effectively obtained [7, 8, 9, 10, 11].

The materials which are hard to cut in modern time are mainly titanium, cobalt, nickel base materials.

The titanium base material is mainly applied to engine fan blade, fan, low, split compressor case and others. Its strength obviously decreases when the temperature is higher than 400–500 °C. Nickel base material is mainly applied to motor combustor, high pressure turbine, and high pressure compressor and so on. The study uses nickel base material Waspaloy to do cutting research. While Waspaloy has good tensile mechanical properties under high-temperature working environment, resistance to fatigue, creep strength, resistance to corrosion, excellent weldability, good tenacity and so on. So it is of used where the high temperature resistance and high load are required. Super-alloy has been seen as the material that is hard to cut in the cutting processing, the main reasons of which are as the follows:

(1) The work-hardening character: the tipping and abrasion of the blade end in the clearance plane between cutting tools increases, and the cuttings is tough and not easy to break off, and the disposal is hard.
(2) Low heat conductivity: for general steel cutting, although it will cause cutting heat which is usually brought away more or less by the cuttings, for super-alloy, it has low heat conductivity, which is easy to accumulate the cutting heat between cutting tool and machined parts. Additionally, the yield point and tensile strength of super-alloy are very high, and cutting impedance is large, so the cutting blade end is easy to cause high pressure, high temperature, and the deformation of cutting tool [22].
(3) The affinity between cutting and super-alloy: for the discontinuous cuttings such as milling, melting phenomenon may exist between the blade end and the cuttings. And the melting object embedding into the workpiece can cause huge impact force, even break the tool [22].

## 2 Materials & methods

### 2.1 Material characteristics

Recently, many nickel base materials are applied to vacuum induction melting technology. The Inconel-718 and Waspaloy are formed through extrusion, and the materials are homogenized through annealing. The purpose of which is to eliminate the segregation or banded structure of crystal structure in the materials. The crystal structure of super-alloy is high-temperature stable FCC Austenitic structure. The structures of crystal phase are as follows Table 1:

**Table 1:** Waspaloy alloy structure.

Form	Categories
Primary Phase	γ: FCC matrix of Ni
Secondary Phases	γ': Ni_3_Al or Ni_3_Ti (FCC) γ": Ni_3_Nb (Tetragonal BCT) δ: Ni_3_Nb (Orthorhombic) η: Ni_3_Ti (HCP)
Carbides	MC, M_23_C_6_, M_6_C and M_7_C_3_

In the nickel base materials, Waspaloy is another kind of precipitation-hardening alloy. The $\gamma^{\prime }$ phase's precipitation hardening is different from that of Inconel-718. The $\gamma^{\prime }$ phase's precipitation hardening is made up of Ti and Al. In addition, Waspaloy alloy has elements which are grain boundary hardened by Carbon, boron and zirconium. In the $\gamma^{\prime }$ phase, the precipitates of Ni_3_Al and Ni_3_Ti can harden the combination of elements. The main hardening sources of Waspaloy are the coherent precipitation hardening of $\gamma^{\prime }$ and $\gamma^{\prime \prime }$ phase. Among them, γ〞is the main hardening phase. Using the parameter control of hot isostatic pressing process makes γ〞phase gain abundant and even distribution [16, 17]. Also, the rise temperature of hot isostatic pressing melts δ phase, indirectly increasing the quantity of precipitates of hardening phase to improve Waspaloy's mechanical strength, As shown in Fig. 3 [18, 19, 20].

**Fig. 2:**
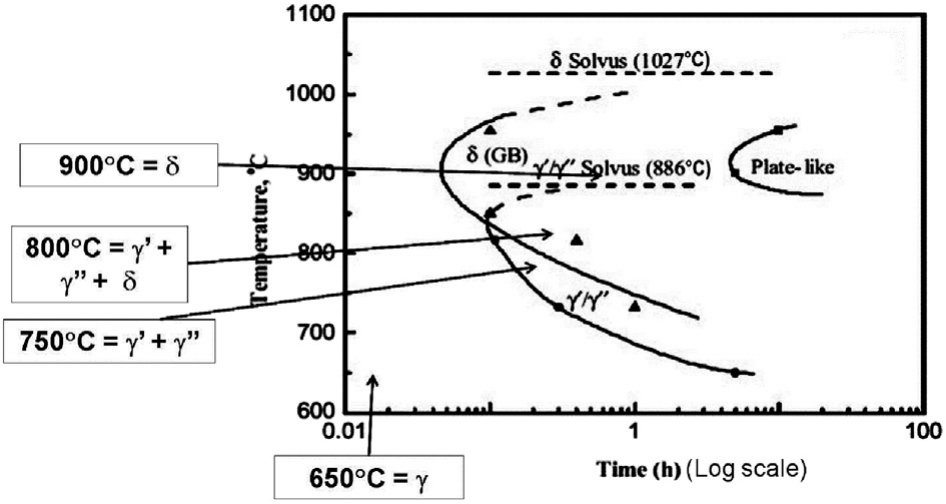
Nickel base super alloy's Time-Temperature-Transformation (TTT).

**Fig. 3:**
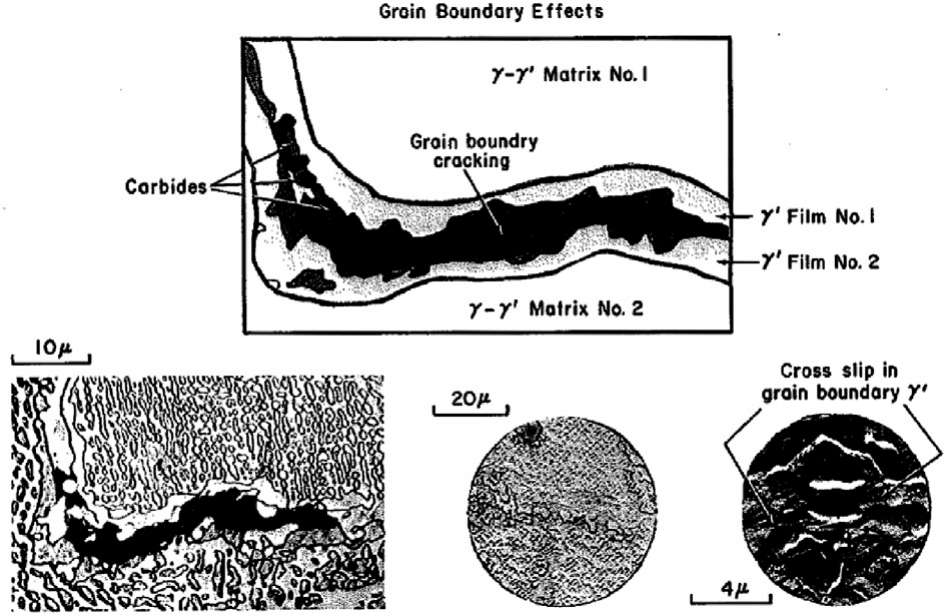
Flows and fracture behavior in the grain boundary of Waspaloy [20].

**Fig. 4:**
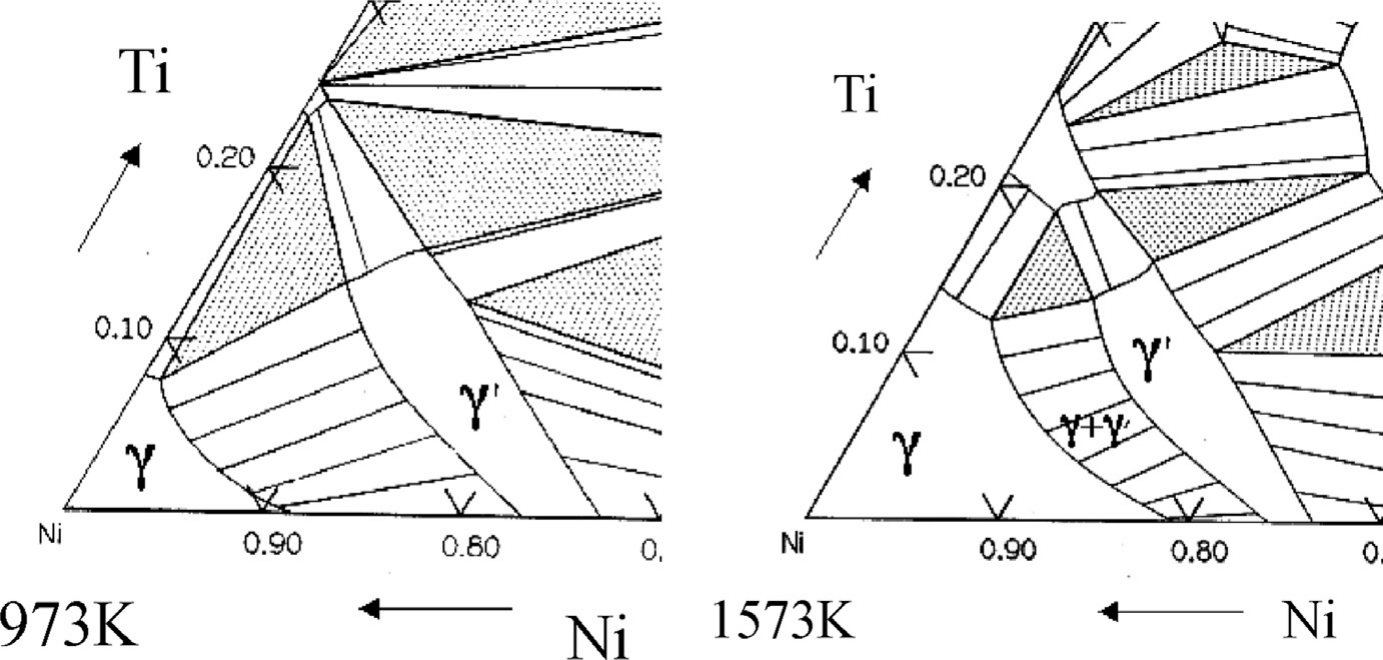
The Ni-Al-Ti ternary phase diagrams show the γ and γ′ phase field [21].

Observe the nickel base super alloy's Time-Temperature-Transformation (TTT), as shown in Fig. 2. The precipitate temperature in the δ phase is higher than that in the $\gamma^{\prime \prime }$ phase, and for the long-time temperature change, δ phase and $\gamma^{\prime }$ phase are smaller than$\gamma^{\prime \prime }$ phase, mainly due to the high-temperature strength of $\gamma^{\prime }$ phase in the high temperature area causes the ability to resist creep deformation. In the $\gamma^{\prime }$ phase, the formation quantity mainly depends on the chemical components and temperature, as shown in Fig. 3. γ phase is mainly the FCC lattice and randomly distributed solute atom, $\gamma^{\prime }$ phase mainly the original cubic lattice, and the Ni atom focuses on the central area, while Al or Ti atoms are on the corner area, As shown in Fig. 4 [14, 15, 21].

Waspaloy of super alloy is one of the aerospace material developed in modern time, The main material properties are as follows (1) The different radius and base materials of solute hardening elements, such as W, Mo, Co, Cr and V, cause part of lattice strain to harden material; (2) Precipitation hardening elements, such as Al, Ti, Nb and Ta, can form coherent and ordered A_3_B type IMC, such as Ni_3_(Al,Ti) and hardening phase (γ′), making the alloy get effective hardening and higher temperature than iron base high temperature alloy and cobalt base alloy; (3) grain boundary strengthening elements, such as B, Zr, Mg and rare earth elements can strength alloy's high temperature property. Table 2 is the component elements of Waspaloy, the standard nickel base material. Therefore, they have hardening precipitation, high affinity and low coefficient of heat conduction phenomenon [2, 13, 17]. How machinability a metal is depends on the physical properties as well as the cutting conditions. Super-alloy waspaloy is more difficult to machinability than some of the other metals due to work-hardening, low heat conductivity and affinity characteristic [5].

**Table 2:** Chemical composition of Waspaloy super alloy.

	Cr	Ni	Co	Mo	W	Ti	Al	Fe	C	Nb	Other
Inconel-718	18	B	0	2.8	0	1	0.5	18	0.025	5.4	
Waspaloy	19.4	B	13.3	4.3	0	3	1.5	2Max	0.035	0	

### 2.2 Study principle and method

#### 2.2.1 Cutting theory

**Fig. 5:**
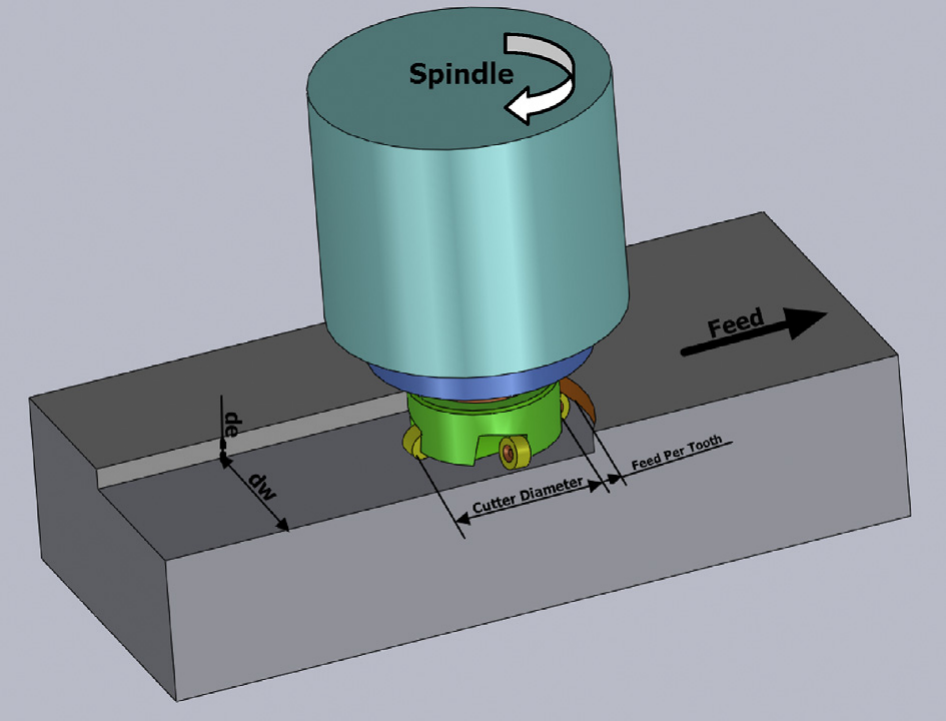
Cutting parameters diagram.

Not only milling is related to the factors of machine tool, cutting tool and fixtures, but also the surface quality and processing efficiency of the workpiece, such as cutting speed, feed rate, cutting depth and width. Different cutting conditions present different results. In addition to the processing quality, the workable valid time also will be affected. Generally, cutting speed, feed rate, cutting depth and width are the cutting conditions set by NC designers. The feed speed has the greatest impact effect on the surface quality, as shown in Fig. 5.

If the cutting efficiency is to be improved, then increase the feed rate. But from the empirical formula of surface roughness, surface roughness is up to the feed speed and the radius of cutting tool's nose end. So increasing the feed speed will cause the surface quality become rough.

#### 2.2.2 Methodology of cutting tool life

The abrasion of cutting tool mainly are wear and breaking, the order of which is wear first and then chipping, and last breaking. The main reasons for wear are 1. the friction between cuttings and tool face, 2. the BUE of the cutting edge, 3. the friction caused by the crack of nose, 4. wear-resistance at high temperature of nose and edge, 5. diffusing effect. The main reasons for breaking are 1. not enough strength of nose to bear cutting resistance, 2. not enough tenacity, 3. the cutting materials can be cut equivalently, 4. cutting temperature caused hardening and other kinds of breaking. To avoid abrasion in the processing of spaceflight materials, the excessive load or normal abrasion causing breaking due to the improper cutting conditions must be taken into consideration, the machining time required for the tool to reach flank wear, for uniform wear is 0.3 mm and non-uniform wear is 0.6 mm (tool life criterion), this study is based on the ISO-8688-1/1994 standard for testing and analysis. While during the processing of nickel base material, precipitates hardening and diffusing affinity cause fast abrasion to the cutting tool. The better the heat resistance, the bigger the n value is. The wear of cutting tool is closely linked to the processing removal. The processing removal volume is proportional to the feed speed, cutting depth and speed. Therefore, Taylor's formula of cutting tool can be corrected as follows:

(5) $$VT^{n}=C$$

When $C={C^{\prime }}^{\frac{1}{s}}F_{t}^{-\frac{e}{s}}dp^{-\frac{d}{s}}$, and $n=\frac{1}{s}$

(6) $$T=C^{\prime }V^{-s}F_{t}^{-e}dp^{-d}$$

In the formula, *Ft* is feed per tooth, dp is feed depth. According to the experiment, the cutting tool life is most sensitive to the speed change, and least sensitive to the cutting depth change, therefore [4]:

(7) s>e>d

In the experimental analysis of RSM, assume that the input parameters that influence the response value y are x1, x2, .., xp, x refers to the independent variable, the unknown function (dependent variable) is:

(8) $$y=f\left(x_{1},x_{2},..,x_{p}\right)+\varepsilon$$

There into, ε is the error (experimental error) of response variable y, if use $E\left(y\right)=f\left(x_{1},x_{2},..,x_{p}\right)=\eta$to express the response of expectancy value, the surface f stands for is response surface, then:

(9) $$\eta =f\left(x_{1},x_{2},..,x_{p}\right)$$

The experiment results solve the constant s, e, d, and establish tool life forecast formula. In order to linearize the tool life formula, use natural logarithm function, Formula (6) to transform, as follows:

(10) $$\ln\;T=\ln\;C^{\prime }+\left|s\right|\ln\;V+\left|e\right|\ln F_{t}+\left|d\right|\ln\;dp$$

When x_0_ = 1, if one response can be imitated by a linear function of an independent variable, the approximate function is called First-Order Model, then x1, x2, x3 are the logarithmic transformations of cutting speed, feed speed and cutting depth, while β0, β1, β2 and β3 are the parameters required to be solved, the model are as shown in Formula (11):

(11) $$y=\beta_{0}+\beta_{1}x_{1}+\beta_{2}x_{2}+...+\beta_{p}x_{p}+\varepsilon$$

y_1_ is the forecast response of first-order model, is proportional to the experiment result y with natural logarithm function, ε is the experiment error value. Value b corresponds to the forecast value of parameter β. Its model is as shown in Formula (12):

(12) $$y_{1}=y-\varepsilon =b_{0}+b_{1}x_{1}+b_{2}x_{2}+b_{3}x_{3}$$

If curvature exists in the tool life forecast system, then higher-order multinomial must be adopted, such as Second-Order Model, the approximate function of which can be expressed as follows:

(13) $$y=\beta_{0}+\sum_{i=1}^{p}\beta_{i}x_{i}+\sum_{i=1}^{p}\beta_{ii}x_{i}^{2}+\sum_{i<j}\sum \beta_{ij}x_{i}x_{j}+\varepsilon$$

**Fig. 6:**
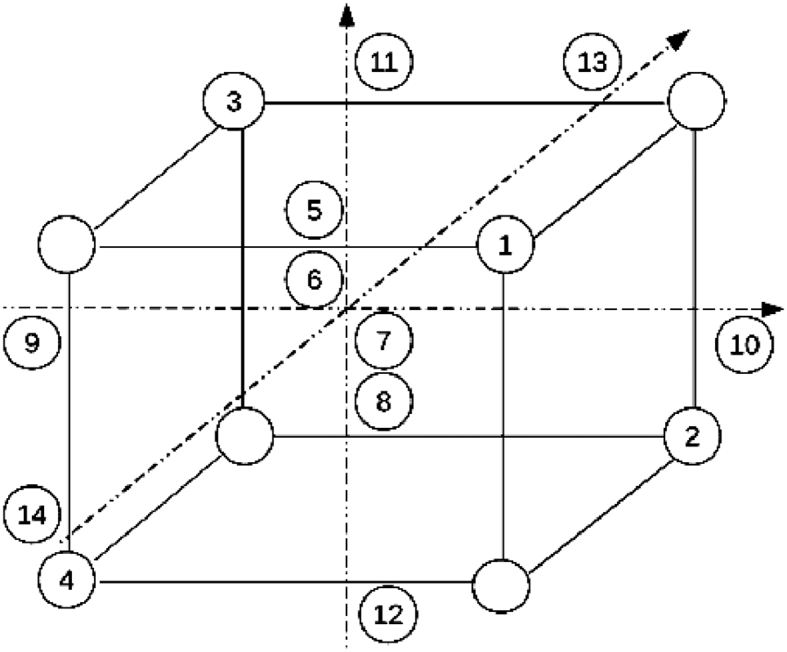
Small central composite design.

RSM is a sequential procedure. When response surface is far from the best condition point, as shown in Fig. 6, only a few curvatures are in the system. It is optimal to adopt first-order model, and the target is to move to near the best point at the speed as fast as possible, using Method of Steepest Ascent to solve. Method of steepest ascent is a procedure to increase move along with the biggest response variable. Once near the best point, adopt second-order model to analyze and find out best point. Usually, central composite design (CCD) is used. CCD is kind of two-level total or part factors design, increased by a few treatment numbers, making the forecast of second-order respond surface model possible.

#### 2.2.3 Experimental facilities and parameters

##### 2.2.3.1 Experimental facilities

**Fig. 7:**
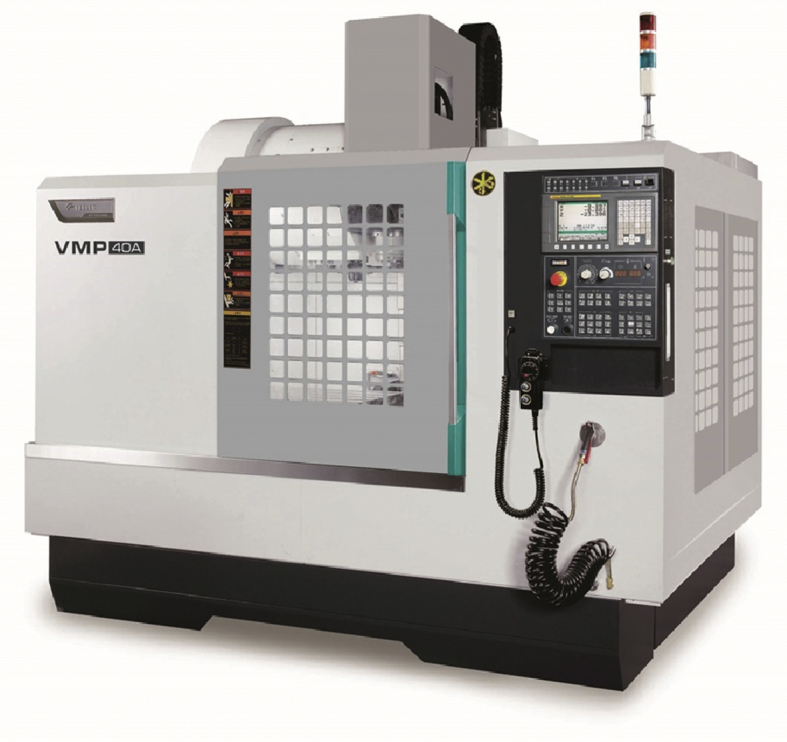
FFG VMP-40(A) machine center.

**Fig. 8:**
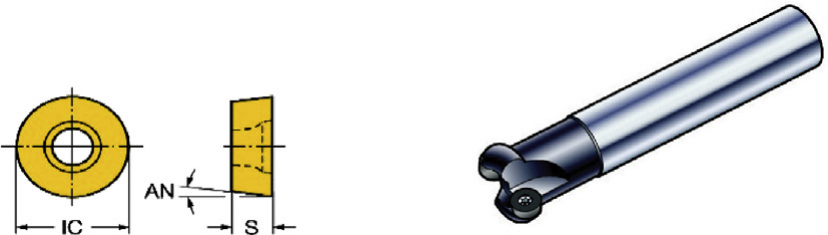
The insert blades and tool-holders.

The experimental use equipment FFG VMP-40(A) machine center to study the milling, as shown in Fig. 7, the experimental insert blades and tool-holders are as shown in Fig. 8 that the equipment are as listed in Table 3. For cutting conditions, they are mainly cutting speed, feed speed, cutting depth, etc. The uppermost aim to set factors and level table is to gain most useful data in the most economical and effective method with the current facilities, and then analyze the optimal data through further statistic concept.

**Table 3:** Experimental equipment.

Equipment name	Specification and type
FFG VMP-40(A) Machine center	X/Y/Z axis travel:1020/520/505mm Maximum speed of revolution: 10000rpm
Insert blades SANDVIK (RCKT1204M-MM2040)	Diameter of blade:12mm R angle of blade:6mm
Milling tool-holders SANDVIK (R200-024A32-16M)	Cutter Diameter:40mm Quantity of insert blade: 3 insert blades (this study adopts one blade)

##### 2.2.3.2 Experimental parameter setting and process

There are 4 steps to analyze for the parameter setting, whose descriptions are as follows:

**Step 1.** Decision of experiment design:

This study is mainly full factorial design. With the properties of central point and axis point, it is central composite design, mainly to improve the precision and correct value of experiment.

**Step 2.** Establishment of experimental conditions:

The tool life test mainly aims at the establishment of significant factors, cutting speed, feed speed and cutting depth and other key factors, to analyze and establish. Based on the design method, add the central point and axis point at α = 1.633. Its factor and level table of experimental axis point can be expressed as follows are as follows Table 4:

**Table 4:** Experiment parameter setting.

Factor name	-1.633	-1	0	+1	+1.633
Cutting speed (m/min)	26.84	30	35	40	43.17
Cutting depth (mm)	0.038	0.1	0.2	0.3	0.36
Feed per tooth (mm/t)	0.038	0.1	0.2	0.3	0.36

**Step 3.** Experimental verification

Carry out cutting experiment, aim at tool wear, and analyze the experiment. Mainly use full factors and central composite design axis point to arrange matrix. The factor values of the variables shown in Table 4 for use in Eqs. (12) and (13) were obtained from the following transforming equations for shown as follows:

$$x_{1}=\frac{\ln\;V-\ln\;35}{\ln\;40-\ln\;35};x_{2}=\frac{\ln F_{t}-\ln\;0.2}{\ln\;0.3-\ln\;0.2};x_{3}=\frac{\ln\;dp-\ln\;0.2}{\ln\;0.3-\ln\;0.2}$$

**Step 4.** Data statistic and analysis

Analysis steps of variance:

(1) Total variance can be divided into experimental factor variance and error variance.
(2) Corresponding degree of freedom determining each variance.
(3) The sum of the squares of variance becomes variance.
(4) Get the statistic of F.
(5) When F > Fα (νR, νE), it means the corresponding response value of R factor is most significant;
(6) When F < Fα (νR, νE), the effect of R factor is not significant.

## 3 Results and discussion

**Fig. 9:**
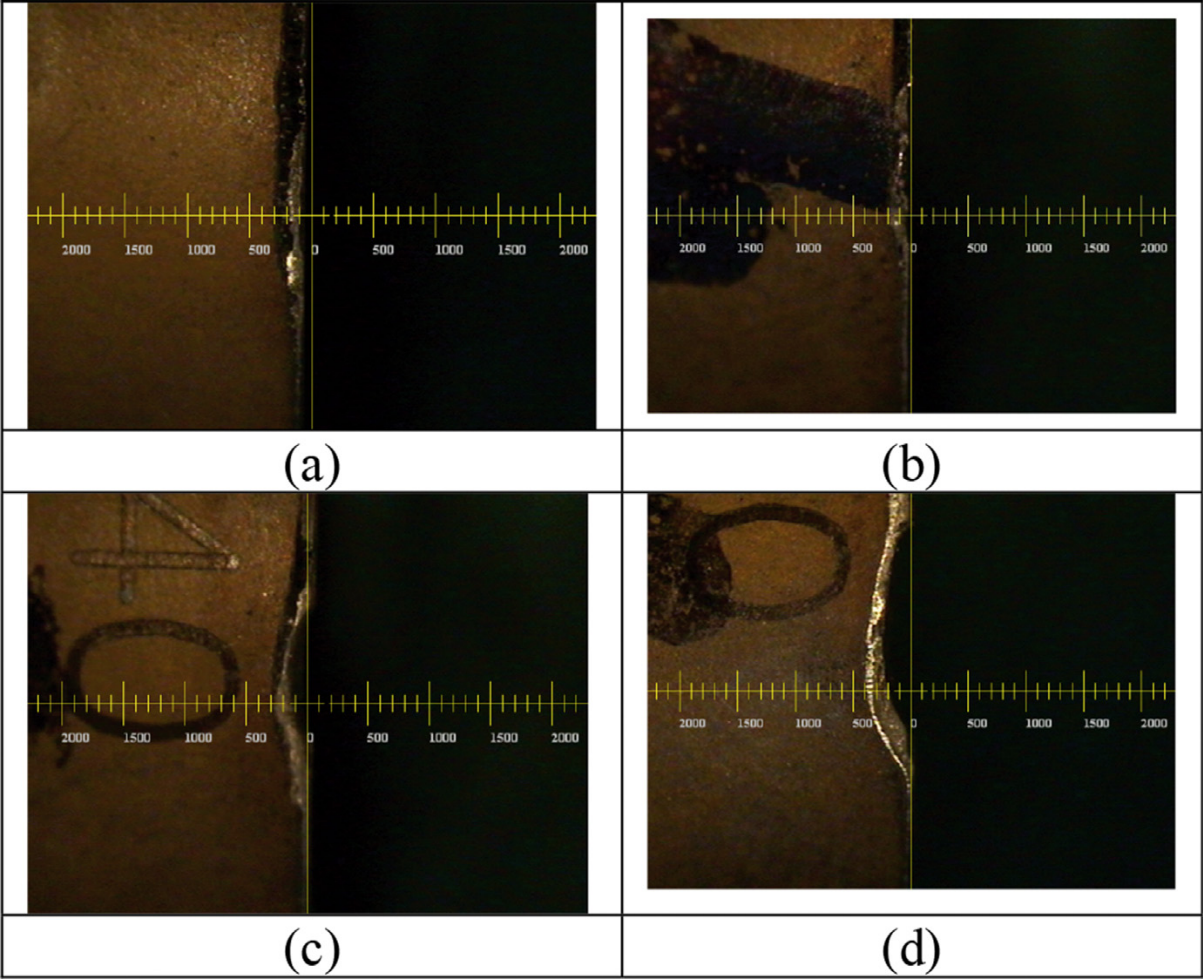
An experimental diagram of tool wear in (a) Ft = 0.1mm/tooth, Dp = 0.1mm,Vc = 30 m/min; (b) Ft = 0.3mm/tooth, Dp = 0.3mm,Vc = 30 m/min; (c) Ft = 0.3mm/tooth, Dp = 0.3mm,Vc = 40 m/min; (d) Ft = 0.2mm/tooth, Dp = 0.2mm,Vc = 43 m/min.

The study mainly analyzes the relation between processing parameters and tool wear through the super-alloy Waspaloy, using the relation between regression analysis method and tool life to establish, as shown in Fig. 9(a) (b) (c) (d). In the tool wear situation, it can be see that when the cutting speed is c = 30 m/min, different feed and cutting depths have no significant wear on the tool, as shown in Fig. 9 (a) (b), when the feed and cutting depths are the same, the cutting speed increases from Vc = 30 m/min to Vc = 40 m/min, as shown in Fig. 9 (b) (c), and the tool wear is significant. The main reason is the material work-hardening, low heat conductivity and affinity characteristic.

### 3.1 First-order model of tool life

In the first-order model, the cutting speed, feed rate of each blade and cutting depth are the main variable factors. While during the process of cutting super-alloy Waspaloy, feed rate of each blade has slight effect on the tool wear. On the given in Table 5, when P-Value <0.05, it is a significant factor. When P-Value = 0.790, it means it is not significant factor in the linear model. In lack-of-fit test of fixed variables, P = * of lack-of-fit test, it can be known that forecast model lacks of fit, which may be pure error (repeated data) and data sub setting (partial data concentrated), therefore, eliminate the nonsignificant factor Ft and analyze again.

**Table 5:** Variance analysis of first-order regression.

Term	Coef	SE Coef	T	P
**Constant**	0.261062	0.01263	20.665	0
**Block**	0.006437	0.01263	0.510	0.618
**V**	0.049274	0.01516	3.250	0.005
**Ft**	-0.004117	0.01516	-0.272	**∗0.790**
**Dp**	0.045418	0.01516	2.996	0.009
S = 0.0553551				
PRESS = 0.0914202				
R-Sq = 56.99%		R-Sq(pred) = 14.45%		R-Sq(adj) = 45.52%

After eliminating the feed rate of each blade Ft, P value in the Lack-of-Fit test is 0.299. When P value is larger than α = 0.05, the forecast model is fit. After it, the test aims at the fitness of first-order or second-order model. The lack of fit test is as follows:

*H*_*1*_: inappropriate regression model (Regression model is nonlinear): $F=\frac{SS_{LOF}}{SS_{PE}}<F_{\left(\alpha ,m-p,n-m\right)}$

*H*_*0*_: appropriate regression model (Regression model is not nonlinear):$F=\frac{SS_{LOF}}{SS_{PE}}>F_{\left(\alpha ,m-p,n-m\right)}$

The forecast formula of P value: $F_{partial}=\frac{\left[SS_{E}\left(reduced\right)-SS_{E}\left(full\right)\right]/\left(m-p\right)}{SS_{E}\left(full\right)/\left(n-m\right)}$

Put the value into the formula and calculate. The discriminants are as follows:

$$F=\frac{\left[0.046-0.045\right]/6}{0.045/10}=0.03$$

$$F=\frac{0.003535}{0.002498}=1.415>F_{\left(0.05,6,10\right)}=0.03$$

From the F value test are as follows Table 6, it can be known that *F > F*_*(0.05,6,10)*_. Therefore, the regression model is not nonlinear. When *R* ^*2*^ = *0.567*, according to the variance analysis table, the discriminant result is too low. It is not proper to stand for first-order model, and second-order model may exist. So axis point is added to analyze the variances of second-order model. The following is the forecast formula of first-order regression model:

$$y_{1}=0.175-0.007V-0.454dp$$

**Table 6:** Variance analysis of first-order regression.

Term	Coef	SE Coef	T	P
Constant	0.261062	0.01226	21.291	0
Block	0.006437	0.01226	0.525	0.607
V	0.049274	0.01417	3.349	0.004
Dp	0.045418	0.01417	3.087	0.007
S = 0.0537289/PRESS = 0.0795788				
R-Sq = 56.78%		R-Sq(pred) = 25.53 %		R-Sq(adj) = 48.67%

Analysis of Variance for Wear						
Source	DF	Seq SS	Adj SS	Adj MS	F	P
Blocks	1	0.000796	0.000796	0.000796	0.28	0.607
Regression	2	0.059876	0.059876	0.029938	10.37	0.001
Linear	2	0.059876	0.059876	0.029938	10.37	0.001
Residual Error	16	0.046189	0.046189	0.002887		
Lack-of-Fit	6	0.021212	0.021212	0.003535	1.42	0.299
Pure Error	10	0.024977	0.024977	0.002498		
Total	19	0.106861				

The following is the forecast formula of first-order tool life:

$$T=2090252V^{-4.5}dp^{-1.12}$$

### 3.2 Second-order model of tool life

In the second-order model, all factor combination and level number can fit second-order regression model, as follows:

$$y=\beta_{0}+\sum_{i=1}^{p}\beta_{i}x_{i}+\sum_{i=1}^{p}\beta_{ii}x_{i}^{2}+\sum_{i<j}\sum \beta_{ij}x_{i}x_{j}+\varepsilon$$

Carry out the lack-of-fit test on second-order model, the test type is as follows:

$$H_{0}:y=\beta_{0}+\sum_{i=1}^{p}\beta_{i}x_{i}+\sum_{i=1}^{p}\beta_{ii}x_{i}^{2}+\sum_{i<j}\sum \beta_{ij}x_{i}x_{j}+\varepsilon$$

$$H_{1}:y\neq \beta_{0}+\sum_{i=1}^{p}\beta_{i}x_{i}+\sum_{i=1}^{p}\beta_{ii}x_{i}^{2}+\sum_{i<j}\sum \beta_{ij}x_{i}x_{j}+\varepsilon$$

From the variance analysis, it can be known that the interacted P-Value = 0.052, so Interaction Model is nonsignificant factor. In the lack-of-fit test, P = *of lack-of-fit test, it can be known that forecast model lacks of fit. Therefore, the nonsignificant factor interaction is eliminated to perform analyze again.

In the second-order tool life model are as follows Table 7, after eliminating the feed rate of each blade Ft and interaction, P value in the Lack-of-Fit test is 0.581. When P value is larger than α = 0.05, the forecast model is fit. Then test aiming at the fitness of first-order or second-order model. The lack of fit test is as follows:

**Table 7:** Variance analysis of second-order regression.

Term	Coef	SE Coef	T	P
Constant	0.241596	0.01408	17.157	0
Block	0.006438	0.01100	0.585	0.567
V	0.049274	0.01319	3.734	0.002
dp	0.045418	0.01319	3.442	0.004
V*V	0.029200	0.01319	2.213	0.043
S = 0.0481804/PRESS = 0.0633557				
R-Sq = 67.42%		R-Sq(pred) = 40.71%		R-Sq(adj) = 58.73%

Analysis of Variance for Wear						
Source	DF	Seq SS	Adj SS	Adj MS	F	P
Blocks	1	0.000796	0.000796	0.000796	0.34	0.567
Regressuon	3	0.071245	0.071245	0.023748	10.23	0.001
Linear	2	0.059876	0.059876	0.029938	12.90	0.001
Square	1	0.011368	0.011368	0.011368	4.90	0.043
Residual Error	15	0.034820	0.034820	0.002321		
Lack-of-Fit	5	0.009844	0.009844	0.001969	0.79	0.581
Pure Error	10	0.024977	0.024977	0.002498		
Total	19	0.106861				

The forecast formula of P value:

Put the value into the formula and calculate. The discriminants are as follows:

$$F=\frac{\left[0.035-0.011\right]/5}{0.011/10}=4.363$$

$$F=\frac{0.00197}{0.00245}=0.8<F_{\left(0.05,5,10\right)}=4.363$$

From the F value test, it can be known that F < F(0.05, 5, 10). Therefore, the regression model does not reject null hypothesis. When *R* ^*2*^ = 0.68, according to the variance analysis table, the discriminant result is too excellent. Other nonsignificant interaction and quadric expression are eliminated to add axis point and analyze the variances of second-order model. The following is the forecast formula of second-order regression model:

$$y_{1}=1.236-0.0719V+0.454dp+0.0011V^{2}$$

### 3.3 Minimum wear forecast

**Fig. 10:**
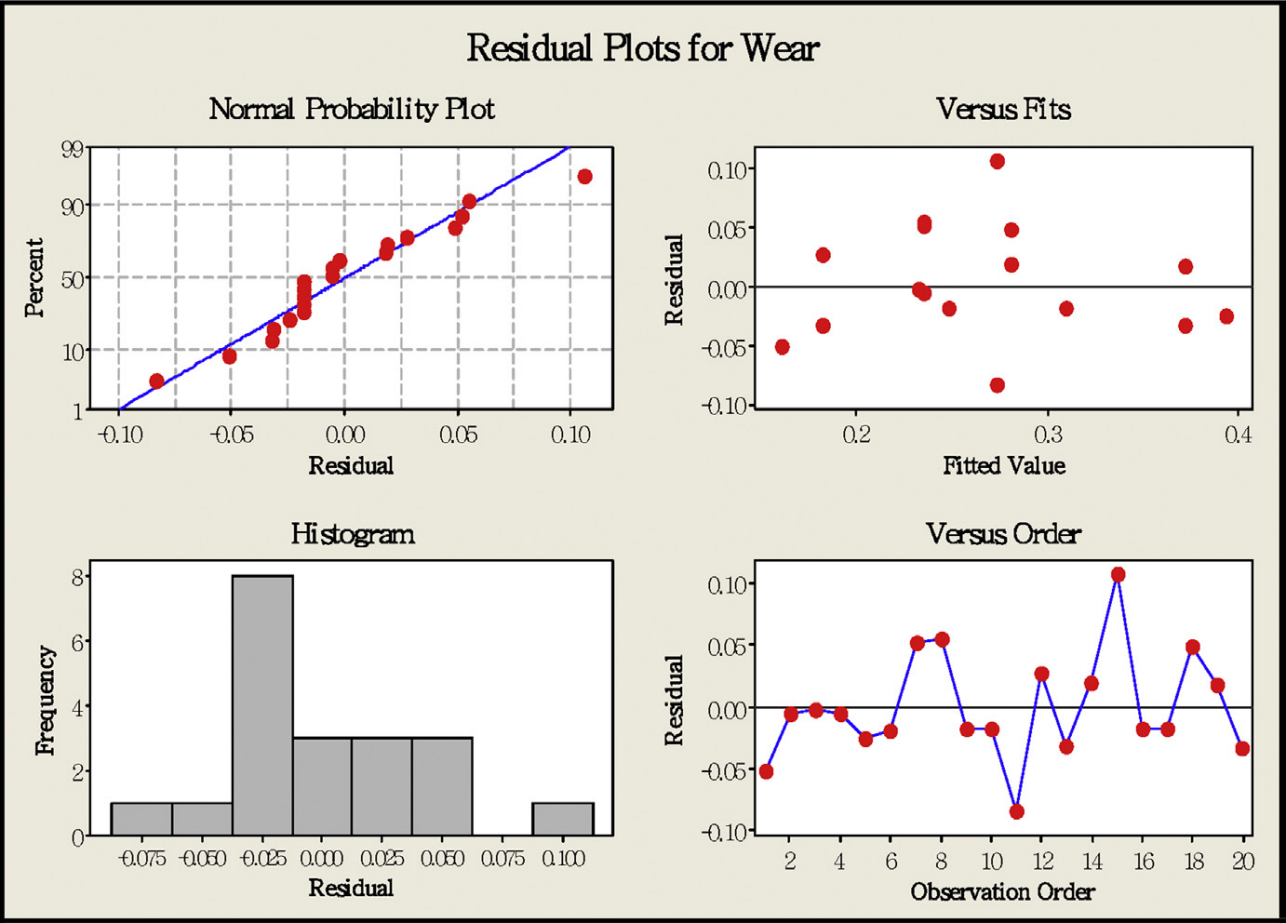
Residual analysis.

In order to improve the precision of tool life forecast formula, the established regression model is used to perform residual analysis and test whether the regression model is appropriate, as shown in Fig. 10. According to the graphical, there is no trend of travel change in violation of assumption. So the configuration model fits the assumption.

**Fig. 11:**
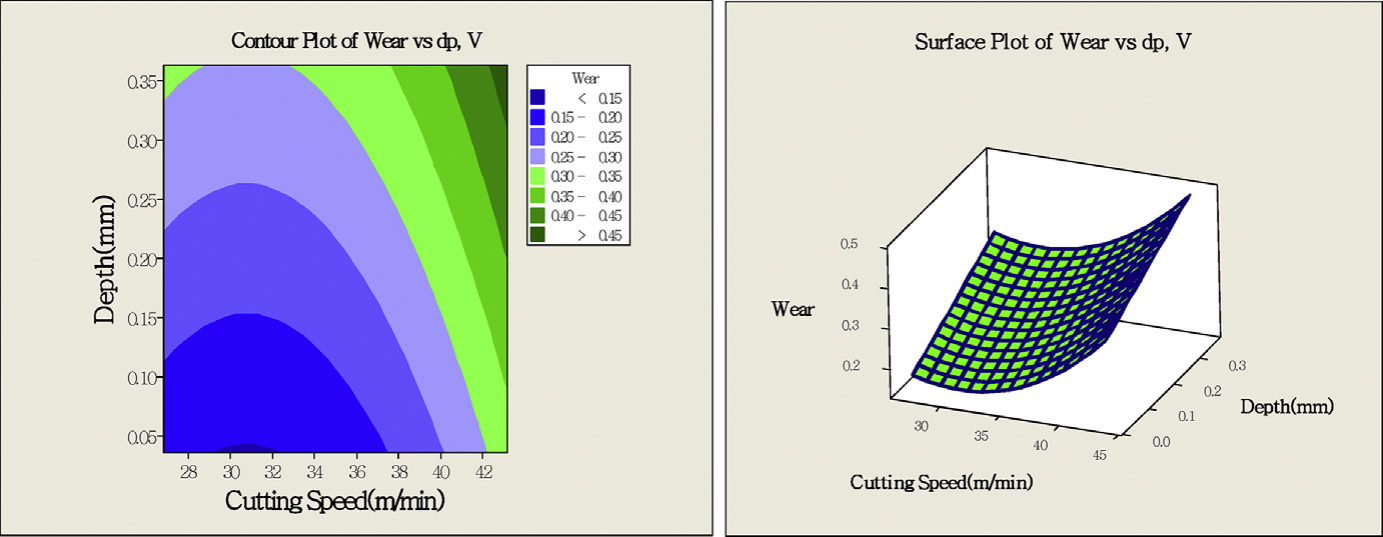
Contour map and response surface map.

When judging the stable point is maximum value, minimum value and saddle point and determining the simplest method on the increasing (decreasing) direction, the fitted model is used to draw contour map and response surface map, and establish the best area. Use proper parameter to find minimum wear. In the second-order tool life model formula, the contour map and 3D response surface map are as shown in Fig. 11. According to the graphical, the cutting speed V = 30～32 m/min is the minimum wear area, and the less the cutting depth, the less the wear is.

**Fig. 12:**
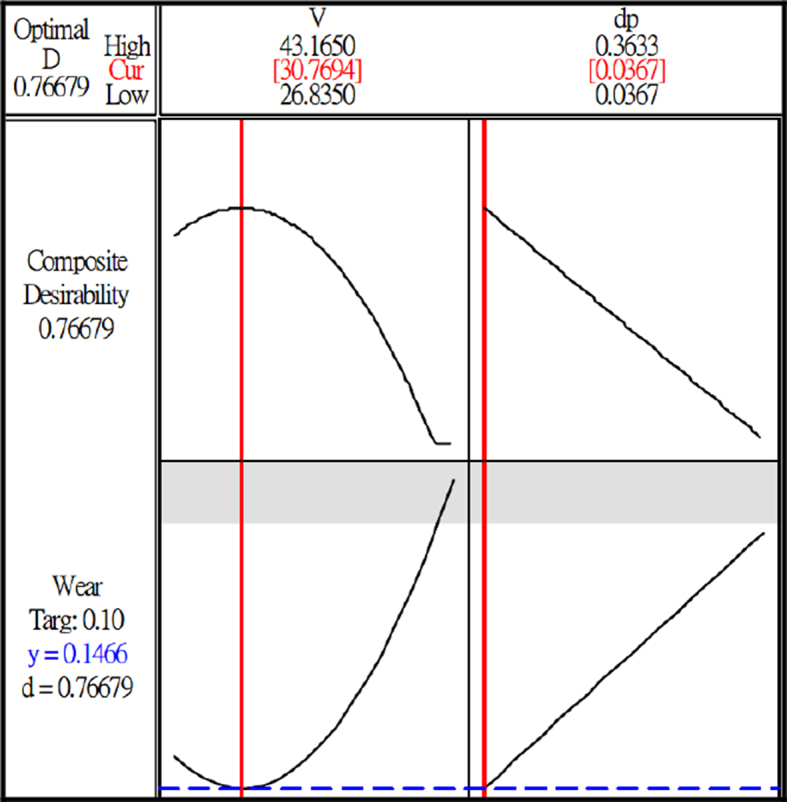
Forecast of minimum tool wear.

**Fig. 13:**
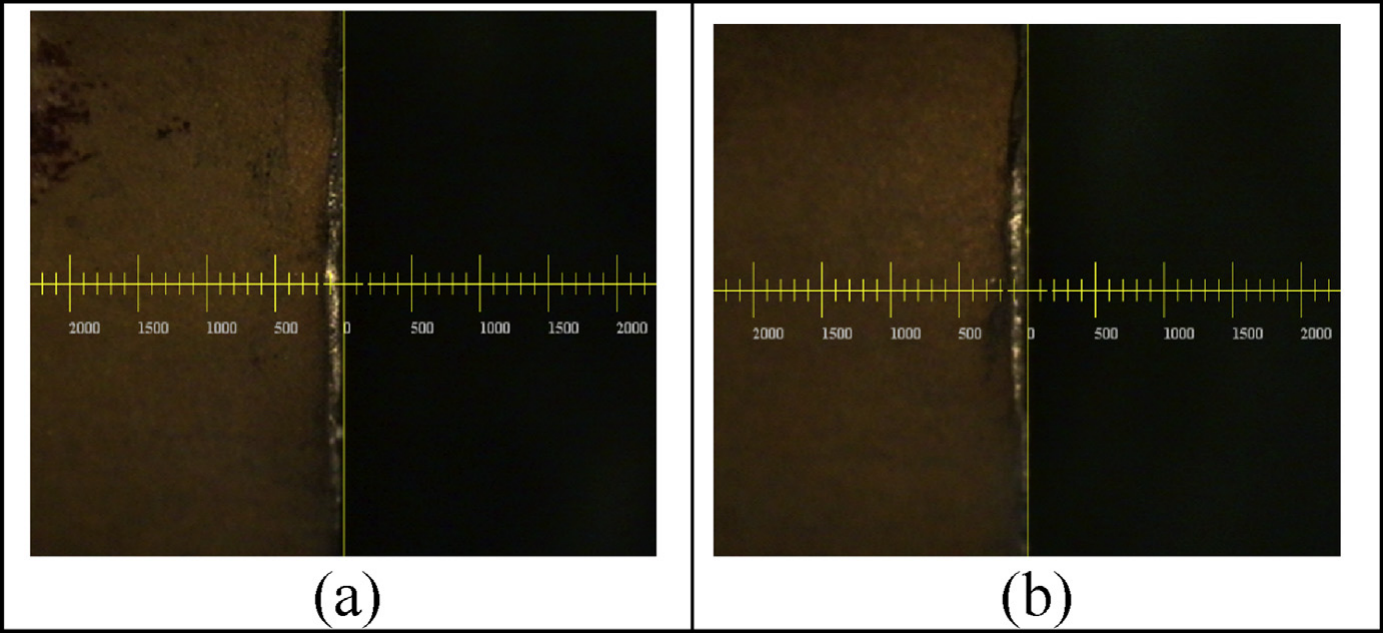
Verification of tool wear.

After analysis, as shown in Fig. 12, when the cutting speed is V = 30.77 m/min and cutting depth is dp = 0.0367mm, the forecast of minimum wear tool can be obtained. Therefore, for long-time cutting, the minimum wear is used to carry out medium processing and finish processing to reach the most ideal condition of tool life. As shown in the Figure, the theoretical predicted value of wear is W = 0.0146mm, the wear after experiment is W = 0.013–0.015mm. In the verification experiments that used three repeated experiments will be performed for analysis and comparison during the verification test, the error after the experiment is less than 15%, as shown in Fig. 13(a) (b).

## 4 Conclusion

1. In the processing of nickel base material Waspaloy, the main influential factors of tool wear are cutting speed and cutting depth. According to the regression analysis method, the cutting speed and depth have little impact on the surface accuracy. Too high cutting temperature causes hardening precipitate, and the affinity of material and tool causes early crack of tool.
2. In the first and second order cutting forecast experiments, the test coefficient is low, mainly because the tool wear during the experiment process is too small, which cannot response and predict, causing the discriminant result is too small. So it easily causes forecast error.
3. This study uses multi-variable tool predictor formula, the main purpose of which is to decrease the error rate. After verification, the error after the experiment is less than 15%. In the processing of nickel base material, material is a very important variable, which easily causes fast tool wear and crack. Therefore, material is one of the important variables in the predictor formula, the theoretical predicted value of wear is W = 0.0146mm, the wear after experiment is W = 0.013–0.015mm. After verification, the error after the experiment is less than 15%.
4. In the same cutting travel, when cutting speed V = 30.77 m/min and cutting depth dp = 0.0367mm, the minimum wear prediction can be obtained.

## Declarations

### Author contribution statement

Shao-Hsien Chen: Conceived and designed the experiments; Performed the experiments; Analyzed and interpreted the data; Contributed reagents, materials, analysis tools or data; Wrote the paper.

Yu-Lun Ho: Performed the experiments; Analyzed and interpreted the data; Contributed reagents, materials, analysis tools or data; Wrote the paper.

### Funding statement

This research did not receive any specific grant from funding agencies in the public, commercial, or not-for-profit sectors.

### Competing interest statement

The authors declare no conflict of interest.

### Additional information

No additional information is available for this paper.

## Symbols

V: Cutting Speed (m/min)
N: Spindle Speed (rev/min)
ØD: Tool Diameter (mm)
F_s_: Feed Rate (mm/min)
T: Insert Number
F_z_: Feed Per Tooth (mm/tooth)

## References

[1] Xue Chao, Chen Wuyi (2011). Adhering layer formation and its effect on the wear of coated carbide tools during turning of a nickel-based alloy. Wear.

[2] Muñoz-Sánchez A., Canteli J.A., Cantero J.L., Miguélez M.H. (2011). Numerical analysis of the tool wear effect in the machining induced residual. Simulat. Model. Pract. Theor..

[3] Hadzley M., Sarah S., Raja Izamshah, Fatin N. (2015). The study of tool wear performance on pecket milling strategy. Appl. Mech. Mater..

[4] Ginta Turnad L., Amin A.K.M. Nurul, Mohd Radzi H.C.D., Lajis Mohd Amri (2009). Tool life prediction by response surface methodology in end milling titanium alloy Ti-6Al-4V using uncoated WC-Co inserts. Eur. J. Sci. Res..

[5] Karaguzel U., Olgun U., Uysal E., Bakkal M. (April 2014). Increasing tool life in machining of difficult-to-cut materials using nonconventional turning processes. Int. J. Adv. Manuf. Technol..

[6] Jeniski R.A., Kennedy R.L. (2005). Development of ATI Allvac^®^ 718Plus^®^ alloy and applications, *ATI 718Plus*^*®*^*alloy background*. Metals Mater. Soc..

[7] Saini A., Chauhan P., Pabla B.S., Dhami S.S. (2016, April 4). Multi-process parameter optimization in face milling of Ti6Al4V alloy using response surface methodology. Proc. IME B J. Eng. Manufact..

[8] Kadirgama K., Noor M.M., Abd Alla Ahmed N. (2010). Response ant colony optimization of end milling surface roughness. Sensors.

[9] Saedona J.B., Soob S.L., Aspinwallb D.K., Barnaclec A., Saada N.H. (2012). Prediction and optimization of tool life in micromilling AISI D2 (∼62HRC) hardened steel. Procedia Eng..

[10] Sidda Reddy B., Suresh Kumar J., Vijaya Kumar Reddy K. (2011). Optimization of surface roughness in CNC end milling using response surface methodology and genetic algorithm. Int. J. Eng. Sci. Technol..

[11] Abou-El-Hossein K.A., Kadirgama K., Hamdi M., Benyounis K.Y. (2007). Prediction of cutting force in end-milling operation of modified AISI P20 tool steel. J. Mater. Process. Technol..

[12] Wagner Vincent, Maher Baili, Dessein Gilles (February 2014). The relationship between the cutting speed, tool wear, and chip formation during Ti-5553 dry cutting. Int. J. Adv. Manuf. Technol..

[13] Dempster Ian, Cao Wei-Di, Kennedy Richard, Bond Betsy, Aurrecoechea Jose, Lipschutz Mark, Loria E.A. (2005). Structure and property comparison of 718Plus alloy and Waspaloy forgings. Superalloys 718, 625, 706 and Derivatives.

[14] Li A., Roberts R., Haldipur P., Margetan F.J., Thompson R.B. (2003). Computational study of grain scattering effects in ultrasonic measurements. Rev. of Prog, in QNDE.

[15] Haldipur P., Margetan F.J., Thompson R.B. (2003). Correlation between local ultrasonic properties and grain size within jet-engine nickel alloy billets.

[16] Stanke F.E., Kino G.S. (March 1984). A unified theory for elastic wave propagation in polycrystalline materials. J. Acoust. Soc. Am..

[17] Haldipur Pranaam, Thompson R. Bruce (2006). Material Characterization of Nickel-Based Superalloys through Ultrasonic Inspection.

[18] Che-Haron C.H., Ginting A., Goh J.H. (2001). Wear of coated and uncoated carbides in turning tool steel. J. Mater. Process. Technol..

[19] Jaffery S.H.I., Mativenga P.T. (2012). Wear mechanisms analysis for turning Ti-6Al-4V-towards the development of suitable tool coatings. Int. J. Adv. Manuf. Technol..

[20] Sims C.T., Hagel W. (1972). The Superalloys.

[21] H. K. D. H. Bhadeshia, Nickel Based Superalloys, University Of CAMBRIDGE.

[22] Polvorosa R., Suárez A., López de Lacalle L.N., Cerrillo I., Wretland A., Veiga F. (April 2017). Tool wear on nickel alloys with different coolant pressures: comparison of Alloy 718 and Waspaloy. J. Manuf. Process..

